# Neonatal Urinary Ascites: A Report of Three Cases

**DOI:** 10.1155/2015/942501

**Published:** 2015-04-14

**Authors:** Adaobi Solarin, Priya Gajjar, Peter Nourse

**Affiliations:** ^1^Department of Paediatrics, Babcock University Teaching Hospital, PMB 21244, Ilishan-Remo, Ogun State, Nigeria; ^2^Renal Unit, Red Cross Children's Hospital, Cape Town, South Africa

## Abstract

Urinary ascites in neonates is not a common condition. Three cases of urinary ascites are presented and each of them has a different aetiology. Neonates with urinary ascites usually present as clinical emergency, requiring resuscitation, ventilator support, and subsequent drainage of urine. The ultimate management depends on the site of extravasation and the underlying cause.

## 1. Introduction

Reports of urinary ascites in the neonates date back to 1681 when Mauriceau [1, 2] gave the earliest description of foetal ascites. It was in 1952 that the first successfully treated case was reported [3]. Although it is a very rare condition, various causes have been attributed. Posterior urethral valve is most common accounting for approximately 70% of the aetiology. It occurs most commonly from the rupture of calyceal fornixes secondary to raised intrarenal pressure. Rarely, urinary bladder perforation is responsible for urinary ascites in posterior urethral valve.

Perforation of the neonatal bladder is rare, with only 17 cases reported between 1956 and 1985 [4]. Predisposing factors apart from posterior urethral valves include neurogenic bladder, congenital bladder diverticulum, and detrusor areflexia [4]. The majority are the results of umbilical arterial catheterization [5] causing rupture of the dome of the bladder or of patent urachus. Spontaneous rupture of the bladder has also been reported in the literature as a result of profound hypoxia or morphine administration [6], though rupture may also occur without clear predisposing factors and is presumably associated with obstructive uropathy, abdominal trauma, neurogenic bladder, difficult obstetric delivery, and iatrogenic injuries during urethral catheterization and umbilical catheterization [7].

Diagnosis is suspected on the basis of ascites with deranged renal function and is confirmed by imaging. Ultrasound helps to establish the presence of ascites and dilatation of the upper tracts with or without associated urinomas and cystic dysplasia of the kidneys. Voiding cystourethrography (VCUG) helps to establish the leak at the level of the urinary bladder by contrast extravasation into the peritoneal cavity and provides information about the underlying disease with associated changes in the urinary tracts. Neonatal urinary ascites is a life-threatening condition as the peritoneal membrane “autodialyzes” the urine, leading to progressive increase in the blood urea nitrogen (BUN) and derangement of the serum electrolytes. Management consists of catheter drainage or surgery depending on the condition of the neonate, with the primary aim of diversion of urine from the peritoneal cavity. Prognosis depends on early diagnosis and adequate urinary drainage.

## 2. Case 1

**Figure 1:**
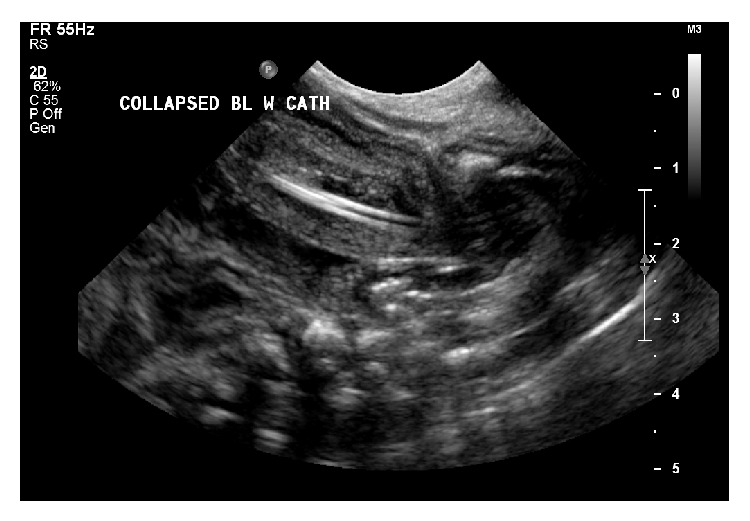
Catheter visualized with tip protruding through the dome of bladder.

Baby S, male preterm, with birth weight of 1800 gm, was delivered at 33-week gestation by SVD on 5/05/2013. He had an Apgar score of 4 in 1 minute, 7 in 5 minutes. He required ventilator support and inotropes. At birth he was oedematous with marked abdominal distension. Mother is blood group O positive and coombs negative; she had apparently normal ultrasound at 16 weeks and 28 weeks. A TORCH screen to identify intrauterine infections was negative. Placental histology showed normal cord vessels and normal membrane while sections showed focal villous oedema. The renal function was deranged with serum creatinine of 148 micromol/L. Ascetic tap performed reveals high creatinine of 249 micromol/L compared to serum creatinine of 148. The baby was anuric; however, urinary catheter was passed and initial output improved to 4 mL/kg/hr. Ultrasonography showed catheter visualized in a collapsed bladder which appears to have a markedly thickened wall. The tip of catheter protrudes through the dome of the bladder (see Figure 1).

Blood creatinine dropped after catheterization from 148 to 42 micromol/L. Baby became polyuric and urinary losses were replaced with appropriate fluid. Cystoscopy and primary repair of posterior bladder wall were done and urinary catheter was removed on day 6 after operation. No posterior urethral valve was identified during the cystoscopy. Urine output normalized to 1 mL/kg/hr. This was a case of an isolated bladder perforation. He has been seen twice on follow-up; his creatinine remains normal, with good stream of urine. He has had one urinary tract infection; isolated organism was* Enterobacter cloacae* which was treated according to sensitivity profile. He has been discharged from urology clinic.

## 3. Case 2

**Figure 2:**
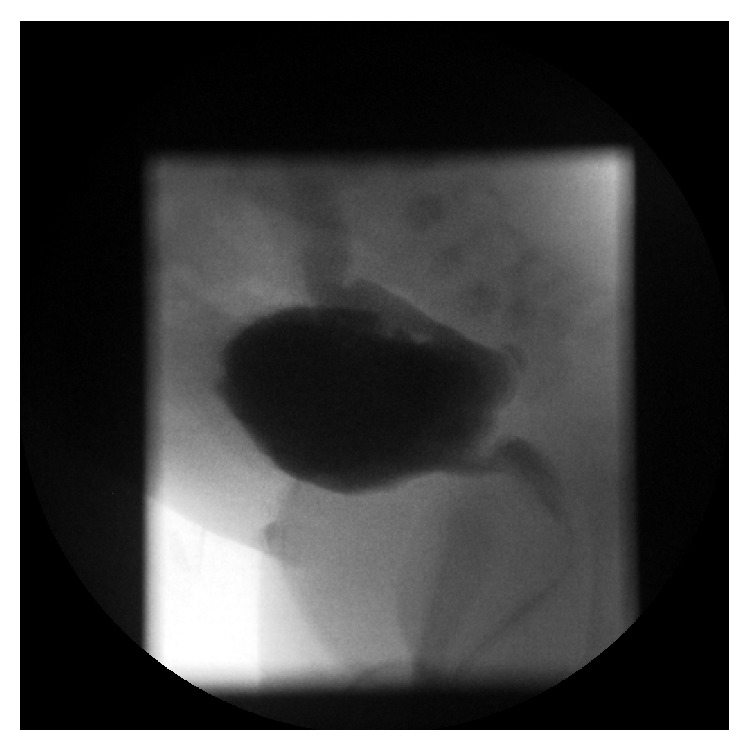
Voiding cystourethrogram confirming the posterior urethral valve.

Baby P, male, with birth weight of 3240 gms, was delivered at 36-week gestation by emergency caesarean section for macrosomia and abdominal ascites on 12/11/2013. Apgar score was 1 in 1 minute, 6 in 5 minutes, and 7 in 10 minutes. He was in marked respiratory distress attributed to the splitting of the diaphragm. There was also severe metabolic acidosis with pH of 6.78, pCO_2_ 17.7, pO_2_ 3.78, base excess of minus 19, and bicarbonate of 9.9. An ultrasound showed multicystic kidneys, marked bilateral hydronephrosis with a thickened bladder wall. Ascites was drained via a Tenckhoff catheterization. He was oliguric; urinary catheterization was done with urine output improving to 1.2 mL/kg/hr. Ascites fluid was analyzed with creatinine level of 118 micrommol/L while serum creatinine was 102 micromol/L. Micturating cystourethrogram showed a posterior urethral valve and a grade 5 vesicoureteric reflux into the right pelvicalyceal collecting system and ureter (see Figure 2). He had the valve ablated on 21/11/13, renal function normalized, and is being followed up by the nephrology and urology teams. He has four proven UTIs (*E*.* coli*,* Klebsiella*,* Enterococcus*, and* Pseudomonas aeruginosa,* resp.) and is treated with appropriate antibiotics according to sensitivity pattern. Renal function remains normal. He had serial ultrasounds done which showed persistent bilateral hydronephrosis and dilated ureters as well as debris in calyces of the left kidney and calcification demonstrated in the lower pole. MAG 3 diuretic renogram showed marked asymmetry of uptake, with left kidney within normal limit. Right kidney was smaller than left one and there was good Lasix response. Later image showed regular, well-defined renal margins after Lasix. Renal output efficiency was similar in both kidneys (left 90%, Rt 88%) and renal relative uptake (Lt 79%, Rt 21%).

He has been booked for reablation on 22/4/2015 by the urology team, and at last follow-up visit 27/02/15 he had no UTI and was between the 25th and 50th centile for weight and at the 50th centile for height.

## 4. Case 3

**Figure 3:**
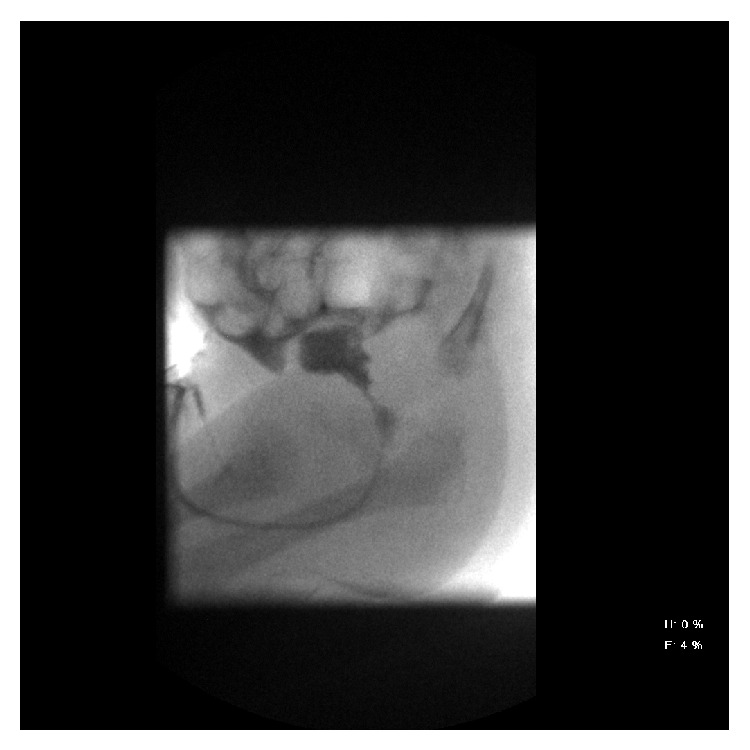
Water-soluble contrast administered via indwelling transurethral catheter. Spontaneous micturition on early filling (approximately 5 mL injected), with immediate extravasation of contrast into the peritoneal cavity. The study was therefore terminated, before the posterior urethra could be adequately evaluated. Bladder rupture is confirmed.

Baby H was delivered by caesarean section at 33 weeks and 6 days of gestation on 23/04/2013. Mother is known with type 2 diabetes mellitus, well controlled on metformin and insulin. Caesarean section was done on account of prolonged rupture of membrane and chorioamnionitis. Baby had respiratory distress at birth, requiring inotropes and ventilator support. He also had* E*.* coli* sepsis and was noted to develop progressively abdominal distension from the third day of life. He had oliguria with impaired renal function and hyponatremia. He was admitted at our center between 20/05/2013 and 26/05/2013. Micturating cystourethrogram showed immediate extravasation of contrast into peritoneal cavity. He had surgical repair of the bladder wall (see Figure 3). Urine output as well as renal function normalized. He was seen by the urology team on 23/08/2013 for follow-up and the renal function remained normal; weight and height are appropriate for age, and he was subsequently discharged from the unit.

## 5. Discussion

All the three cases presented had in common prematurity and hypoxia (see Table 1); however, there was no history of trauma in any of them. One of the cases was a posterior urethral valve. Urinary ascites in posterior urethral valve can occur as a result of rupture of calyceal fornixes or transudation across the intact upper tract. It can also occur following the rupture of bladder wall. In obstructive uropathy, the upper tracts are subjected to high pressures in the intrauterine life. This affects the development of the kidneys and cystic renal dysplasia ensues. However, protective mechanisms do exist to prevent this irreversible damage to the kidneys. These protective mechanisms include vesicoureteral reflux, bladder diverticula, and urine extravasation [8]. Extravasation at the level of the fornixes may result in urinoma formation around the kidneys, which may remain contained or communicate freely with the peritoneal cavity, leading to urinary ascites.

Urinary ascites can also occur following spontaneous or iatrogenic bladder rupture. The prolonged exposure to hypoxia that leads to ischemic visceral damage may also cause ischemic lesions to the bladder, which may lead to the rupture. Vasdev et al. [9] reported urinary ascites following bladder rupture of six cases. They attributed the rupture to hypoxia in all but one case. None of the cases Vasdev et al. [9] described was secondary to trauma and their time of presentation ranged from within 24 hours of life to 24 days. Umbilical arterial catheterization causing trauma to the dome of the bladder or to a patent urachus accounts for 75% of bladder ruptures [10]. The Foley catheter has also been reported to induce rupture [11, 12]. Morphine administration can cause urinary retention, which may lead to the rupture of the bladder [6].

Morrell et al. reported urinary ascites in a female preterm baby who had a spontaneous bladder rupture due to hypoxia [13]. Hypotension, abdominal distention, and electrolyte imbalance were observed in their patient. In this case report, Morrell et al. suggested that nontraumatic rupture of the bladder occurred as a result of hypoxia and hypotension, signifying that the bladder fundus vascular circulation is sensitive to ischemia [13]. The other case report in the literature described two preterm babies with nonobstructive urinary bladder rupture. This time, hypotension, severe respiratory distress, and umbilical artery catheterization were presented as risk factors [14]. Hypotonia and a full-stretched bladder may facilitate necrosis and spontaneous rupture of the bladder due to pressure [10].

Two of our presented cases had urinary ascites following bladder rupture. These babies had hypoxia and hypotension (requiring inotropes) and were also preterm. These are part of the identified risk factors for urinary ascites. Vasdev et al. [9] in their report highlighted the histologic appearance of the bladder following rupture. They postulated possible fetal hypoxia as reason for the ischaemic insult to the bladder. However, in our first patient who was born hydropic, histology of the placenta was morphologically abnormal while that histology of the debrided bladder was normal. This may be explained by the possibility that hypoxia could have occurred during delivery and not in utero.

The current diagnostic workup of patients with neonatal ascites consists of ultrasonography and paracentesis. The biochemical analysis of the ascites fluid should confirm the diagnosis of its origin; creatinine, urea nitrogen, and potassium concentrations are higher than the plasma levels [15]. In our cases, simultaneous plasma and ascites chemistry showed that creatinine concentrations of the ascites fluid were higher than the plasma creatinine concentrations, indicating urinary ascites in all cases. Findings on USG include hydroureteronephrosis, urinomas, ascites, changes in bladder in the form of thickening of the bladder wall, diverticula, and dilated posterior urethra.

VCUG is the modality of choice for demonstration of anatomy of the lower urinary tract, allowing confirmation of diagnosis of PUV, changes in the bladder, vesicoureteral reflux (VUR), and actual extravasation of the contrast from a rent in the bladder wall. Bladder changes for long-standing urethral obstruction include a large capacity or a contracted bladder with trabeculation, sacculation, or diverticula. There may be associated bladder neck hypertrophy seen as narrowing at the bladder neck.

Management has to be prompt with the basic aim of achieving decompression of the urinary tract. This may be accomplished by abdominal paracentesis, catheter drainage, or surgical exploration and repair of the bladder wall. One of the indications of abdominal paracentesis includes respiratory distress [16]. In cases of PUV, catheter drainage by urethral route with or without vesicostomy achieves healing in most patients in 10–14 days. Catheter drainage fails in ruptures with large rents and requires surgical repair. Our patients with identified rupture of the bladder were surgically repaired. Prognosis depends on the age at diagnosis and the extent of changes in the urinary tract.

A high degree of suspicion for urine leak as a cause of ascites when there is no clear cause of ascites should always be entertained. Once a baby without urethral obstruction has urinary ascites, this usually indicates a large bladder defect that will not close spontaneously with catheter drainage.

## Figures and Tables

**Table 1:** Summary of the various cases and the management.

Cases (patients)	1	2	3
Gestational ages (weeks)	33	36	33

Birth weight (grams)	1800	3240	2500

Apgar score at 1, 5, and 10 mins or asphyxiated with abnormal blood gas	4, 7	1, 6, and 7	Asphyxiated

Ventilator support	Yes	Yes	Yes

Inotropes	Yes	No	Yes

Deranged renal function	Yes	Yes	Yes

Time of presentation of bladder rupture following birth	At birth	At birth	72 hrs

U/S, VCUG	Urethral catheter seen piercing dome of bladder	Bilateral hydronephrosis, hydroureters, and thickened trabeculae bladder. PUV is confirmed on VCUG	MCUG revealed extravasation of contrast into peritoneal cavity

Identifiable causes	Hypoxia, hypotension, and prematurity	PUV, hypoxia, and prematurity	Hypoxia, hypotension, and prematurity

Management of the bladder rupture	Abdominal paracentesis. Surgical repair of bladder wall tear	Conservative management. Abdominal paracentesis. Urethral catheter in situ for 10–14 days. Ablation of valve	Surgical repair of bladder wall tear

Clinical outcome	Successful	Successful, on long term follow-up	Successful
